# A Comparison of the Functional Outcome in PHILOS (Proximal Humerus Internal Locking System) Versus Proximal Humerus Interlocking Nail in Proximal Humerus Fractures

**DOI:** 10.7759/cureus.74617

**Published:** 2024-11-27

**Authors:** Karuna Shankar Dinkar, Amrit Goyal, Vivek Mittal, Rajat Kapoor, Hemant S Chahar, Vikas K Singh

**Affiliations:** 1 Orthopaedics, Sarojini Naidu Medical College, Agra, IND; 2 Orthopaedic Surgery, Sarojini Naidu Medical College, Agra, IND; 3 Orthopaedics, Chahar Fracture Polyclinic, Agra, IND

**Keywords:** constant-murley score, deltopectoral approach, philos, proximal humerus fracture, proximal humerus nail

## Abstract

Background

Proximal humerus fractures (PHFs) are often injuries that occur in the field of orthopedics and can either be treated conservatively or operatively. The PHILOS (Proximal Humerus Interlocking System) and the proximal humerus nail (PHN) are the commonly used operative techniques.

Objectives

The objective of this study is to analyse the functional outcome of the PHILOS (Proximal Humerus Internal Locking System) and the PHN (Proximal Humerus Nail) in proximal humerus fractures.

Methods

This research included a total of 40 patients; out of them, 20 patients were assigned to the PHN group, while the other 20 patients were assigned to the PHILOS group. Functional outcomes were evaluated using established scoring methods, such as the Constant-Murley Shoulder Outcome score and the Disabilities of the Arm, Shoulder and Hand (DASH) questionnaire.

Results

Following the inclusion criteria, a total of 40 patients were found to be eligible for the study. The PHN group had a significantly reduced average surgical time of 75.90 minutes (±5.70 SD) compared to the PHILOS group, which had an average surgical time of 112.50 minutes (±12.93 SD). The average constant score at 12 months follow-up was 72.70±3.51 for PHILOS and 78.35±4.72 for PHN group (p-value<0.001).

Conclusion

Our study indicates that PHN yields favorable functional outcomes in the treatment of PHFs as compared to PHILOS. Nevertheless, more investigation, such as prospective randomized controlled trials (RCTs) and extended follow-up, is necessary to validate these results.

## Introduction

Proximal humerus fractures (PHFs) are becoming more common due to the rapid increase in population, and they make up about 6% of all fractures in the human body [1]. These fractures often occur in elderly individuals in the osteoporotic bones after a fall at ground level while extending their arm. Furthermore, given the aging of the general population and rising prevalence of decreased bone density among elderly individuals, there is a significant emphasis on developing both nonoperative and surgical strategies for PHF treatment. A majority of these fractures may be managed conservatively, including the utilization of a sling to immobilize the injured region and subsequent participation in activities to regain functionality [2]. Nevertheless, to achieve favourable functional outcomes, surgical intervention is necessary for fractures that are displaced and unstable. According to studies, these fractures comprise around 12.6% of all fractures in the proximal humerus [3]. Various methods have been documented for treating these fractures including non-surgical realignment, surgical realignment followed by fixation using bone stitches, tension bands, wires placed around the bone, nails inserted into the bone, or plates with special locking mechanisms, as well as the use of artificial joint replacements [4]. Several problems have been documented, including the occurrence of screws and plates being dislodged or loosened, nonunion, avascular necrosis, and the development of rotator cuff impingement syndrome. The selection of the surgical technique mostly relies on the patient's age, their preferred clinical outcomes, and ability to participate in and adhere to the rehabilitation regimen. The goal is to quickly achieve stability and near-perfect anatomical alignment in order to allow for immediate deployment. Nailing has various benefits over plating, including its minimally invasive nature with small skin incisions, less harm to soft tissues, and the ability to accommodate larger implants. Nevertheless, there is a higher probability of malunion and open reduction carries the risk of infection and harm to the soft tissues. Surgeons consider delays in surgical procedures detrimental since it increases the difficulty of accomplishing reduction and may lead to the absorption of cancellous bone. The controversy about the most effective approach to treating proximal humerus fractures continues, despite the progress made in orthopedic surgery procedures. The Proximal Humerus Internal Locking System (PHILOS) and Proximal Humerus Interlocking Nail (PHN) are widely used techniques for treating proximal humerus fractures; however, comprehensive research comparing their effectiveness remains limited. The objective of this research is to analyse and compare the clinical and functional outcomes of PHILOS and PHN in patients with PHFs.

## Materials and methods

**Figure 1:**
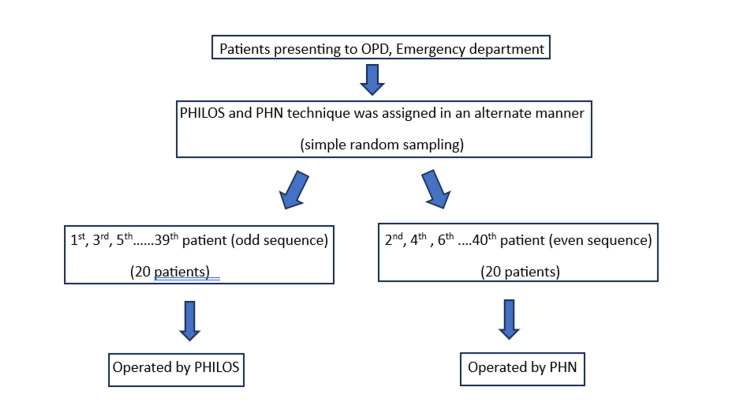
Flowchart illustrating the selection of patients through simple random sampling

This prospective comparative study was conducted on 40 patients who were registered in either the emergency department or the outpatient department (OPD) of the Sarojini Naidu Medical College Hospital from October 2022 to March 2024. Prior to their inclusion in this research, written informed consent was obtained from the participants in their native language, and the study received ethical approval from the Institutional Ethics Committee of the Sarojini Naidu Medical College, Agra (SNMC/IEC/2024/264). A sample of 40 patients who met certain criteria for having proximal humerus fractures were randomly chosen for this study (Figure 1). Out of these patients, 20 had surgery using the PHILOS locking plate, whereas the other 20 underwent surgery using the PHN. Inclusion criteria were as follows: (1) patients aged 18-75 years involving both sexes; (2) two-part proximal humerus fracture; (3) closed proximal humerus fracture; (4) patients consenting to study; (5) extraarticular PHFs. The exclusion criteria were as follows: (1) all open fractures; (2) refusal to provide informed consent; (3) medically unfit patients; (4) patients aged beyond 18-75 years; (5) intraarticular proximal humerus fractures; (6) infected nonunion, and (7) pathological fractures.

Surgical procedure

**Figure 2:**
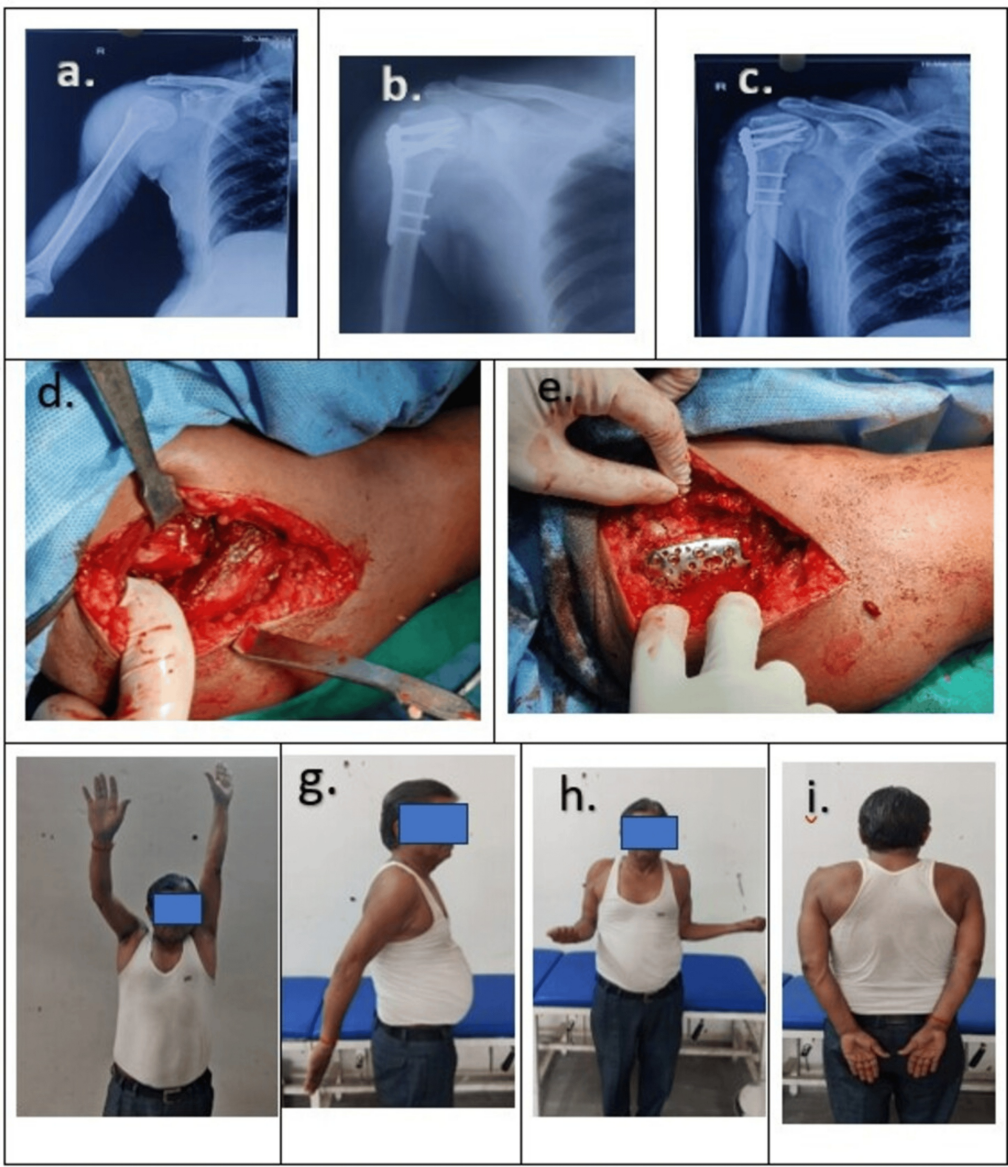
Patient with PHF treated by PHILOS plate (a) Preoperative X-ray of the right shoulder joint; (b) postoperative X-ray; (c) six months follow-up X-ray; (d) opening of fracture by deltopectoral approach; (e) final fixation of the PHILOS plate; (f, g, h, i) functional outcome PHF: Proximal Humerus Fractures; PHILOS: Proximal Humerus Internal Locking System

The patients underwent surgery on a radiolucent operating table where all procedures were performed by the same team, skilled in both techniques. In the plate group, the deltopectoral approach was used in all cases (Figure 2). After exposing the fracture, reduction was achieved and confirmed using fluoroscopy. The plate was then positioned and secured.

**Figure 3:**
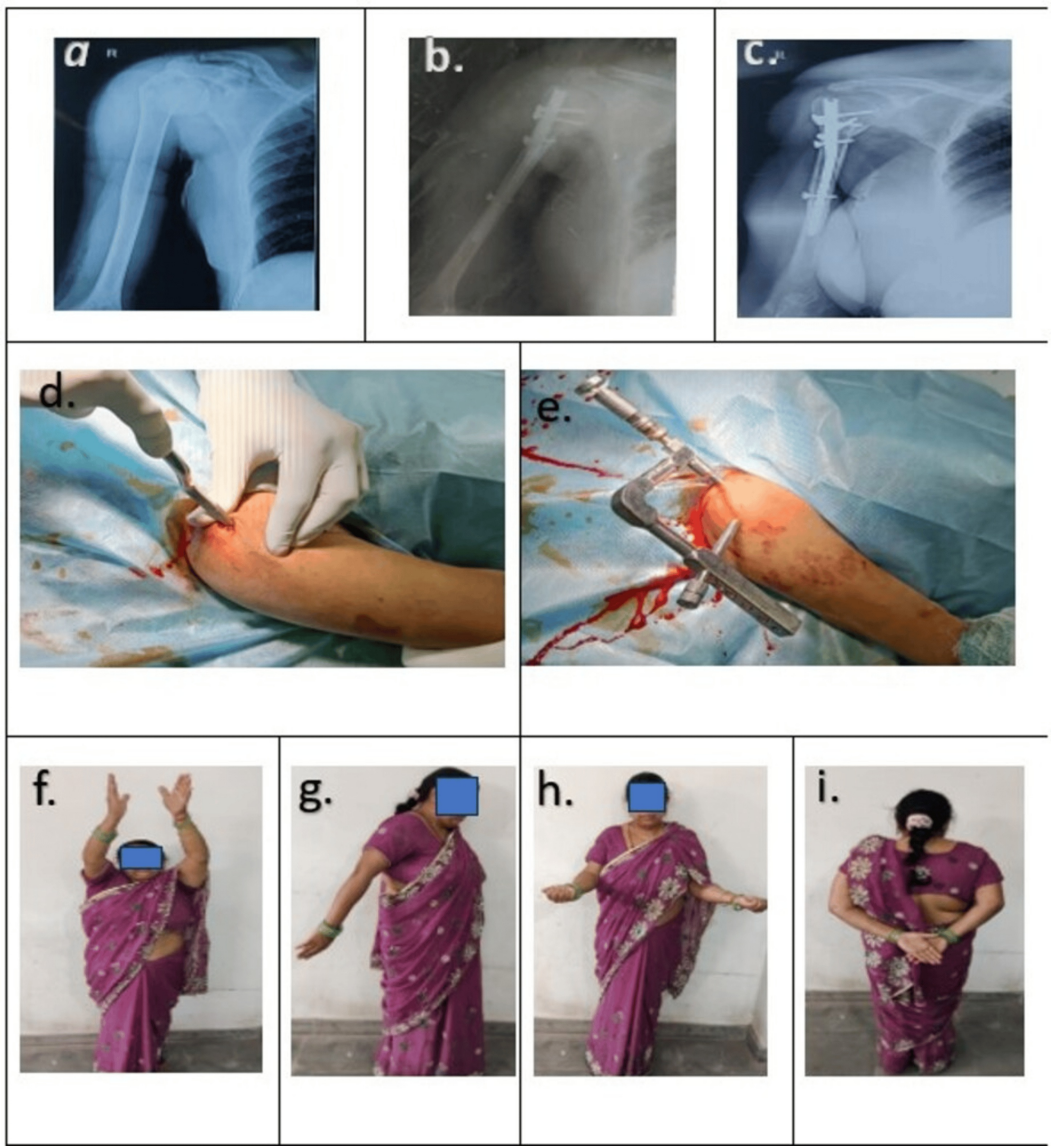
Patient with PHF treated by PHN (a) Preoperative X-ray of the right shoulder joint; (b) postoperative X-ray; (c) six months follow-up X-ray; (d) entry made with bone awl; (e) proximal locking done through jig with the help of a sleeve; (f, g, h, i) functional outcome PHF: Proximal Humerus Fracture; PHN: Proximal Humerus Interlocking Nail

For the nail group, a small incision was made along the deltoid muscle fibres to expose the subdeltoid bursa (Figure 3). The supraspinatus tendon was partially incised, and the entry point was identified at the highest point of the humeral head under fluoroscopy, aligned with the bone shaft. A bone awl was used to make the entry point, and a guide wire was inserted and aligned with the humeral diaphysis under fluoroscopic guidance. After reaming the entry hole, the nail was advanced over the guide wire. Proximal locking screws were inserted percutaneously through drill sleeves using a jig, followed by distal screws in the same manner. The jig was then removed, and final orthogonal imaging was performed.

Clinical and radiographic data were collected for all patients, including age, gender, mechanism of injury, comorbidities, operation time, and blood loss. Functional outcomes were assessed using the Constant-Murley Shoulder Outcome score and Disabilities of the Arm, Shoulder and Hand (DASH) score. Postoperatively, the arm was immobilized with a sling for six weeks. Passive range of motion exercises began the next day if the reduction was stable. Active exercises were initiated at six weeks, with strength and resistance training introduced between 10 and 12 weeks.

## Results

An equal number of patients (n=20) were enrolled in this PHILOS group and the PHN group. There were 14 males (70.0%) and six females (30.0%) in the group operated with the PHILOS plate while in the PHN group, there were 12 males (60.0%) and eight females (40.0%). The mean age of the participants was 50.00±13.25 years in the PHILOS group and 54. ±11.53 years in the PHN group. The PHILOS group had an average age of 50.00 years (±13.25 SD), while the PHN group had an average age of 54.85 years (±11.53 SD). In the PHILOS group, 70% of the fractures were on the left and 30% on the right. In the PHN group, 60% of fractures are on the left and 40% on the right. Fractures treated with the PHILOS system were caused by falls on the ground in 85% of cases and by road traffic accidents (RTA) in 15% of the patients. For the PHN group, 75% of fractures resulted from falls, while 25% were attributed to RTAs. In addition, the initial characteristics of the patients in both groups were comparable (Table 1).

**Table 1:** Comparison of baseline characteristics of the patients between PHILOS group and the PHN group using ‘t’ or chi-square test Data has been represented as mean ± SD for baseline characteristics and n denotes sample size for each group where p value <0.05 considered significant, n and % = number and percentage of variables of baseline characteristics in each group. PHILOS: Proximal Humerus Internal Locking System; PHN: Proximal Humerus Interlocking Nail; RTA: road traffic accidents.

		PHILOS group (n=20)		PHN group (n=20)		t	p-Value
		Mean	±SD	Mean	±SD		
Age (years)	-	50.00	13.25	54.85	11.53	-1.22	0.230”
		n	%	n	%	Chi Sq.	p-Value
Gender	Male	14	70.0	12	60.0	0.11	0.740
	Female	6	30.0	8	40.0		
Side	Left	14	70.0	12	60.0	0.11	0.740
	Right	6	30.0	8	40.0		
Mode of Injury	Fall On Ground	17	85.0	15	75.0	0.16	0.693
	RTA	3	15.0	5	25.0		

The mean operation time was 112.50±12.93 minutes in the PHILOS group and 75.90±5.70 minutes in the PHN group. The mean blood loss was 201.45±8.89 ml in the PHILOS group and 103.00±8.65 ml in the PHN group. In addition, the mean operation time and blood loss (ml) were significantly higher in the PHILOS group compared to the PHN group. The mean DASH score before surgery, after three months, six months, and 12 months respectively was 68.10±2.53, 54.15±2.87, 45.80±2.93 and 38.55±3.22 in the PHILOS group and 67.55±3.03, 52.35±3.79, 42.30±4.26 and 29.55±3.39 in the PHN group. The mean DASH score was comparable between the groups before surgery and at three months, while it was significantly higher in the PHILOS group compared to the PHN group at six and 12 months. In addition, the DASH score was significantly lower in both the PHILOS and the PHN groups from preoperative to 12-month follow-up (Table 2).

**Table 2:** Association of mean DASH Score between PHILOS group and PHN group at pre-op, three months, six months, and 12 months using ‘t’ test Data has been represented as mean±SD for DASH score where p-value <0.05 considered significant, n=sample size in each group. DASH: Disabilities of the Arm, Shoulder and Hand;  PHILOS: Proximal Humerus Internal Locking System; PHN: Proximal Humerus Interlocking Nail

DASH Score	PHILOS group (n=20) (Mean±SD)	PHN group (n=20) (Mean±SD)	t	p-Value
Pre-OP	68.10±2.53	67.55±3.03	0.62	0.537
3 months	54.15±2.87	52.35±3.79	1.69	0.098
6 months	45.80±2.93	42.30±4.26	3.03	0.004
12 months	38.55±3.22	29.55±3.39	8.60	<0.001
p-Value	<0.001	<0.001		

The mean Constant-Murley Shoulder Outcome score before surgery, after three months, six months and 12 months was respectively 34.40±2.70, 47.30±2.77, 58.40±2.84 and 72.70±3.51 in the PHILOS group and 35.10±2.13, 50.50±4.19, 64.65±4.51 and 78.35±4.72 in the PHN group. The mean Constant-Murley score was comparable between the groups before surgery, while it was significantly higher in the PHN group compared to the PHILOS group at three months, six months and 12 months. In addition, the Constant-Murley score was significantly higher in both the PHILOS group and the PHN group from preoperative to 12-month follow-up (Table 3).

**Table 3:** Association of mean constant score between the PHILOS group and PHN group at pre-op, three months, six months, and 12 months using ‘t’ test. Data has been represented as Mean ± SD for constant score where p value <0.05 considered significant, N= sample size in each group

Constant-Murley score	PHILOS group (n=20) (Mean±SD)	PHN group (n=20) (Mean±SD)			t	p-Value
Pre-OP	34.40±2.70	35.10±2.13			-0.91	0.368
3 months	47.30±2.77	50.50±4.19			-2.85	0.007
6 months	58.40±2.84	64.65±4.51			-5.25	<0.001
12 months	72.70±3.51	78.35±4.72			-4.30	<0.001
p-Value	<0.001		<0.001

In the PHILOS group, 0.0% of the screws loosened, compared to 10.0% in the PHN group. Acromion impingement syndrome was 5.0% in the PHN group and 10.0% in the PHILOS group. There were 0.0% superficial infections in the PHN group and 10.0% in the PHILOS group. Aseptic necrosis and malunion/nonunion were not found in either group. In terms of complications, both groups were comparable (Table 4).

**Table 4:** Association of different complications between the PHILOS group and the PHN group using chi-square test. n= sample size in each group, n and % = number and percentage of individuals showing particular complication in each group respectively where p value <0.05 considered significant. PHILOS: Proximal Humerus Internal Locking System; PHN: Proximal Humerus Interlocking Nail

Complications	PHILOS group (n=20)		PHN group (n=20)		Chi-square	p-Value
	n	%	n	%		
Screw backout	0	0.0	2	10.0	0.56	0.468
Acromion impingement syndrome	2	10.0	1	5.0	0.36	0.548
Superficial infection	2	10.0	0	0.0	0.56	0.468
Aseptic necrosis	0	0.0	0	0.0	-	-
Malunion/nonunion	0	0.0	0	0.0	-	-

The radiological assessments during follow-up focused on several factors, including the quality of fracture reduction, alignment, restoration of articular congruity, fracture union, and any issues with the PHILOS plate and PHN such as deviation or screw penetration. All fractures successfully united. Radiographic evidence of bone union for PHN group was 3.1±1.6 months and 3.3±1.3 months for the PHILOS group showing shorter union time for PHN group, but the difference among the two groups was not statistically significant (p>0.05). There were no instances of implant deviation, screw penetration, screw backout, impingement, or implant failure.

## Discussion

Advancements have been made in the treatment of proximal humerus fractures. Although some of the injuries require surgical intervention, the majority can be effectively managed through nonoperative methods. When considering several surgical choices, it is important to identify the procedure that provides better results in terms of the extent of movement, alleviation of pain, occurrence of complications, and general functioning of the shoulder. This comparison provided a framework for clinical decision-making, resulting in the establishment of uniform treatment regimens and improved patient care.

In our study, we enrolled 20 patients in two groups: PHILOS and PHN. There were 14 male participants (70.0%) and six female participants (30.0%) in the group treated with the PHILOS plate. In the proximal humeral nail (PHN) group, there were 12 male patients (60.0%) and eight female patients (40.0%). The PHILOS group had a mean age of 50.00 years, while the PHN group had an average age of 54.85 years. The PHILOS group had 70% left-side and 30% right-side fractures, while the PHN group had 60% left-side and 40% right-side fractures. PHILOS injuries were 85% due to falls and 15% due to RTA, while PHN injuries were 75% due to falls and 25% due to RTA. Both groups had comparable initial characteristics. Our study showed that the mean operation time was 112.50 ±12.93 minutes in the PHILOS group and 75.90±5.70 minutes in the PHN group, which clearly shows that the mean operation time was significantly higher in the PHILOS group compared to the PHN group. According to Edwards et al. (2006), nail fixation is faster than plate fixation. Nail insertion is less invasive and reduces the complexity and time required for surgery [5]. Sharma et al. (2019) believe PHN surgery may take less time than PHILOS plating. This efficiency is due to the less invasive insertion of the nail, which reduces surgical complexity and soft tissue exposure. These data suggest that PHN may improve the surgical efficiency of proximal humeral fracture treatment and patient outcomes [6].

In our study, the mean blood loss was 201.45±8.89 ml in the PHILOS group and 103.00±8.65 ml in the PHN group. Blood loss was significantly higher in the PHILOS group compared to the PHN group. Gnanesh et al. (2020) studied functional outcomes following PHN fixation for displaced proximal humerus fractures. While they did not directly report on blood loss, their findings of successful outcomes with PHN imply potential advantages in terms of surgical efficiency and reduced intraoperative complications, including blood loss [7]. Li et al. (2018) found in a meta-analysis of 20 studies that locking plates or intramedullary nailings (IMNs) for PHF repair caused the same postoperative problems and consistent scores. In contrast, IMNs reduced blood loss, fracture healing time, operative time and incision length [8].

Our study showed that the mean constant score was similar before surgery, but the PHN group had a significantly higher score than the PHILOS group at three months, six months and 12 months. The PHILOS and PHN groups also had significantly lower DASH scores from preoperative to 12-month follow-up. Jagiasi et al. (2018) found that PHILOS patients had a mean Constant-Murley score of 61.8, with a significant age difference: 50.53 in those older than 45 years and 72.91 in those younger than 45 years. PHILOS benefits younger patients with higher constant scores [9]. Vijayvargiya et al (2016) found mean constant scores of 70.9 and 74 for the PHILOS plate, delta-splitting and deltopectoral methods, respectively. This study found that the surgical approach influences the outcomes, but PHILOS provides excellent functional results [10]. Jhamnani et al. (2023) found a mean constant score of 85.8 at follow-up. This high score shows that the PHILOS method treats PHF well, while PHN achieved better long-term outcomes in our study [11]. Gradl et al. (2009) note that both treatments work, but our study shows that PHN improves function [12]. Matziolis et al. (2010) found that Zifko nails were superior to locking plate osteosynthesis for two-part proximal humerus fractures [13]. Our excellent results with PHN align with and support these findings. Von Rüden et al. (2014) found comparable functional results, but nailing mobilized faster and had fewer problems [14]. In our study, the mean DASH score was similar before surgery and at three months, but the PHILOS group had a significantly higher score than the PHN group at six and 12 months. The PHILOS and PHN groups also had significantly lower DASH scores from preoperative to 12-month follow-up. Narayanan et al. (2018) found that patients had an average DASH score of 8.69, indicating satisfactory functional recovery after surgery [15]. The authors assessed patients' DASH scores and range of motion. These results support Ismail et al. (2012) [16] and Zu-Bin Zhou et al. (2012) who claim that minimally invasive percutaneous plate osteosynthesis (MIPPO) with locking compression plates improves shoulder range of motion. Zhou et al.'s (2012) range of motion data shows that these techniques improve postoperative shoulder function [17]. Locking compression plates and minimally invasive MIPPO and minimally invasive plate osteosynthesis (MIPO) improved shoulder range of motion and DASH scores in patients with proximal humeral fractures, according to studies by Narayanan et al. [15], Ismail et al. [16], Zhou et al. [17], and Altman et al. [18] . These results emphasize the importance of these treatments for patient satisfaction and function. In our study, screw loosening occurred in 0.0% of the PHN group and 10.0% of the PHN group. The prevalence of acromion impingement syndrome was 5.0% in the PHN group and 10.0% in the PHILOS group. There were no superficial infections in the PHN group, while the PHILOS group had a 10.0% rate of superficial infections. There was no aseptic necrosis or malunion/nonunion in either group.

Our study has several limitations. Firstly, we included only two-part fractures, which may limit the generalizability of the study's results to more complex fracture patterns, thereby restricting its applicability to a broader spectrum of proximal humerus fractures. Secondly, this study focuses solely on functional outcomes and excludes radiological assessments, which could have provided further insights. Radiological evaluation, such as fracture alignment and implant positioning, might have offered a more complete understanding of the efficacy of PHILOS versus proximal humerus nail. Thirdly, this study has a relatively small sample size, which decreases the generalizability of the findings to the broader population and limits the statistical power to detect clinically significant differences between the two treatment methods.

## Conclusions

The rationale for performing this comparison research is explained by the need for evidence-based recommendations that may provide surgeons with valuable information to enhance their decision-making and ultimately enhance patient outcomes. Overall, while having identical age, gender distribution, and injury modalities, the PHN group had superior functional results compared to the PHILOS group. Additionally, the PHN group experienced less blood loss and shorter operating durations. The PHN group had superior progress in both constant and DASH scores during a 12-month period in comparison to the PHILOS group. Studies have shown that while both implant options are effective for treating proximal humerus fractures, PHN is often seen as the superior choice. Although this is a single-centred study, more diverse study with larger sample sizes and longer follow-up periods is needed to validate these findings.
